# Blocking the PCNA/NKp44 Checkpoint to Stimulate NK Cell Responses to Multiple Myeloma

**DOI:** 10.3390/ijms23094717

**Published:** 2022-04-25

**Authors:** Muhammed Iraqi, Avishay Edri, Yariv Greenshpan, Oron Goldstein, Noa Ofir, Priyanka Bolel, Muhammad Abu Ahmad, Miri Zektser, Kerry S. Campbell, Ory Rouvio, Roi Gazit, Angel Porgador

**Affiliations:** 1 The Shraga Segal Department of Microbiology, Immunology, and Genetics, Faculty of Health Science, Ben-Gurion University of the Negev, Beer Sheva 8410501, Israel; iraqi@post.bgu.ac.il (M.I.); edriavi@post.bgu.ac.il (A.E.); yarivg@post.bgu.ac.il (Y.G.); orongold@post.bgu.ac.il (O.G.); noaof@post.bgu.ac.il (N.O.); bolel@post.bgu.ac.il (P.B.); abuahmam@post.bgu.ac.il (M.A.A.); gazitroi@bgu.ac.il (R.G.); 2 The Shraga Segal Department of Microbiology, Immunology, and Genetics, Faculty of Health Science, National Institute for Biotechnology in the Negev, Ben-Gurion University of the Negev, Beer Sheva 8410501, Israel; 3 Internal Medicine A and Multiple Myeloma Clinic, Soroka Medical Center, Beer Sheva 8489501, Israel; spivakma@bgu.ac.il (M.Z.); rubio@bgu.ac.il (O.R.); 4 Blood Cell Development and Host Defense Program, Research Institute at Fox Chase Cancer Center, Philadelphia, PA 19111, USA; kerry.campbell@fccc.edu

**Keywords:** multiple myeloma, PCNA, immune check point, NKp44, clones

## Abstract

**Simple Summary:**

Membrane-associated PCNA is expressed on the surface of human MM cell lines and primary MM cells. Mab 14-25-9 interacts with membrane-associated PCNA and blocks its binding to NK-expressed NKp44, thus activating NK function. We showed that mAb 14-25-9 can serve as an immune checkpoint blocker, enhancing the function of NK cells on target human MM cell lines and primary cells.

**Abstract:**

Multiple Myeloma (MM) is a devastating malignancy that evades immune destruction using multiple mechanisms. The NKp44 receptor interacts with PCNA (Proliferating Cell Nuclear Antigen) and may inhibit NK cells’ functions. Here we studied in vitro the expression and function of PCNA on MM cells. First, we show that PCNA is present on the cell membrane of five out of six MM cell lines, using novel anti-PCNA mAb developed to recognize membrane-associated PCNA. Next, we stained primary bone marrow (BM) mononuclear cells from MM patients and showed significant staining of membrane-associated PCNA in the fraction of CD38^+^CD138^+^ BM cells that contain the MM cells. Importantly, blocking of the membrane PCNA on MM cells enhanced the activity of NK cells, including IFN-γ-secretion and degranulation. Our results highlight the possible blocking of the NKp44-PCNA immune checkpoint by the mAb 14-25-9 antibody to enhance NK cell responses against MM, providing a novel treatment option.

## 1. Introduction

Natural killer (NK) cells are sentinels of the innate immune system. They provide a critical response against transformed and virus-infected cells that have down-regulated class I MHC (MHC-I) to escape CTL killing [1]. NK cells eliminate target cells through directed exocytosis of perforins, granzymes, and cytokines such as IFN-γ and TNF-α [2]. Both activating and inhibitory receptors regulate NK cells, and the balance of signaling determines tolerance vs. attack of normal or abnormal cells, respectively.

NKp46, NKp44, and NKp30 (also called Natural Cytotoxicity Receptors, NCR1, -2, and -3, respectively) are type 1 transmembrane proteins, which function as major NK cell-activating receptors. Their structure is composed of one or two extracellular domains that bind the ligand and a transmembrane domain that interacts with intracellular signaling proteins [3,4]. The engagement of any of these receptors by tumor cells stimulates cytotoxicity and cytokine production, especially IFN-γ [5,6]. A basic amino acid in their transmembrane domains mediates non-covalent association with a transmembrane acidic residue in an accessory signaling protein; NKp44 associates with DAP12, while NKp30 and NKp46 interact with TCR-ζ and FcεRI-γ [7]. The accessory proteins contain immune-receptor tyrosine-based activation motifs (ITAM), which recruit Syk/ZAP-70 tyrosine kinases [8]. NKp44 is expressed by activated peripheral NK cells [5]. Multiple experiments have shown that NCRs have a key role in the NK cell-mediated lysis of tumor cells; remarkably, the involvement of a given NCR in triggering cytolysis of a distinct transformed target cell varies among cells of the same or different histotype [9]. This implies that the NCRs may recognize distinct ligands, and that transformed cells can express variable surface densities of different NCR ligands [7].

Several ligands for NCRs were reported. We and others have shown that NCRs are involved in the functional recognition of viral hemagglutinins [10,11,12,13,14,15] and various configurations of tumor-membrane-associated heparan sulfates (HS) [16,17,18,19,20]. NKp46 has several virally induced ligands such as hemagglutinin of influenza [14], avian Newcastle disease [12], and vaccinia virus [21]. Other known ligands for NKp46 are vimentin and complement factor P [22]. NKp30 has been reported to recognize the B7H6 ligand [23]. It was also reported to recognize the viral ligand pp65 of CMV (cytomegalovirus) [24] and BAT3 (HLA-B associated transcript 3) [25]. We have previously reported that NKp44 can recognize PCNA (Proliferating Cell Nuclear Antigen) [26]. Interestingly, PCNA is a cancer-associated nuclear factor that exists as a homotrimer to form a clamp around nuclear DNA and promote replication [26].

The increased expression of PCNA is highly noticed in proliferating cells, including cancer cells [27], and it serves as a clinical marker for malignancy [28]. We previously showed that the interaction of the activating receptor NKp44 with target cell expressed PCNA inhibits NK cells’ functionality [26]. While it was initially perplexing how NKp44 could detect the nuclear PCNA in tumor target cells, we further showed that PCNA is present on tumor cell surfaces and is recruited to the NK immunological synapse [26]. Notably, NKp44 has three distinct differentially spliced isoforms. NKp44 isoform 1 uniquely expresses a single cytoplasmic ITIM (immunoreceptor tyrosine-based inhibition motif) that is associated with the inhibitory effect of PCNA on NKp44-mediated function, but the ITIM is absent in isoforms 2 and 3 [29]. Thus, the recognition of PCNA at the surface of tumor cells by splice-isoform 1 of NKp44 serves as a novel immune checkpoint through which tumors can evade immune destruction [30].

To further study PCNA-NKp44 interactions, specific tools are needed. We recently developed a monoclonal antibody (mAb 14-25-9) which has a unique capacity to recognize an epitope of PCNA that is exposed on the cell surface. mAb 14-25-9 effectively blocks the binding of PCNA by NKp44 [31]. Treatment of a variety of tumors with mAb 14-25-9 enhanced NK cell-mediated cytotoxicity in vitro, as well as in PDX models (patient-derived xenograft bearing NSG mice) [32]. Importantly, while our results provide exciting evidence that NKp44-PCNA blocking can potentiate NK cell-mediated anti-tumor responses, the impact of the mAb 14-25-9 on the response of NK cells to distinct cancer types requires further studies. Apart from PCNA, NKp44 has several virally induced ligands such as hemagglutinin of influenza and Sendai viruses or from avian Newcastle disease virus [12,13,15], or envelope glycoproteins from West Nile and Dengue flaviviruses [33]. Other NKp44 ligands are intracellular proteins serving as cell surface ligands such as the activating NKp44L which was first shown to be induced by the HIV-1 envelope protein gp41 on infected CD4^+^ T cells with expression levels increasing with viral load [34]. Recently another novel NKp44 ligand was discovered as PDGF-DD engagement of NKp44 was shown to trigger NK cell activation by Colonna et al. [35].

Multiple myeloma (MM) is a malignant disorder which is characterized by expansion of plasma cells in the bone marrow, accumulation of monoclonal Ig protein in blood and urine, and end-organ damage that may include anemia, bone lesions, renal failure, and hypercalcemia [36]. About 32,270 cases of MM were diagnosed last year in the United States, with an estimated 12,830 fatalities [37]. Over the past decade, new therapeutic regimens have entered clinical practice to treat MM, including immunomodulatory drugs (lenalidomide, pomalidomide), proteasome inhibitors (bortezomib, carfilzomib), and anti-tumor antibodies (daratumumab, elotuzumab). These therapies, in combination with conventional treatments, have significantly improved the overall survival of MM patients over the last decade from a median of approximately 3–4 years to about 5–7 years [38,39,40]. However, over 90% of MM patients relapse after treatment despite progress in therapeutic options; moreover, they develop treatment resistance [41]. Therefore, new therapies are needed to prolong remissions and hopefully bring a cure.

NK cells have multiple activities against myeloma. The graft-versus-myeloma effect has been reported in cases of allogeneic transplantations, demonstrating that relapse rates were affected by the mismatch between NK-inhibitory receptors (KIR) and their MHC-I ligands in the recipient patient [42,43]. Such studies define a major potency for NK cells to prevent MM relapse. Interestingly, NK cells are well known to possess both direct cytotoxicity and Antibody Dependent Cellular Cytotoxicity (ADCC) against MM, ex vivo and in patients [44,45,46,47]. NK activities are induced by activating receptors, including NCRs, NKG2D, and DNAM-1, which have cognate ligands on MM cells [46,47,48]. NKs are also inhibited by numerous inhibitory receptors, and several studies already reported significant alterations in the activating/inhibitory ratio of NK in advanced MM patients [47,49,50,51,52,53,54]. Interestingly, current therapies including the most commonly used melphalan, bortezomib, and lenalidomide, can enhance the NK cytotoxicity thus increasing MM elimination [48,53,54,55,56,57,58,59,60,61]. While many new therapies and combinations of therapies have improved patient survival, MM remains largely incurable as almost all patients relapse and become refractory [62], thus strongly calling for improved activation of NK cytotoxicity within patients.

In the last decades, there has been a greater understanding of the importance of therapeutic monoclonal antibodies. mAb’s are proving to have increased efficacy in the treatment of different human cancers, including non-solid cancers [63,64]. The anti-tumor effects of mAbs can be via multiple mechanisms: blocking receptors, engaging immune cells, inducing ADCC, opsonizing for phagocytosis, and activation of the complement [65]. Some treatments for MM that act by targeting specific cell surface antigens are already in clinical use, including treatments that target signaling lymphocytic activation molecule F7 (SLAMF7), CD38, CD138, CD56, CD200, CD40, BCMA, and CD74 [66,67,68,69,70,71,72,73]. We and others have demonstrated that the FDA-approved antibodies elotuzumab, which targets SLAMF7, and daratumumab, which targets CD38, function at least in part by promoting NK-cell mediated ADCC [74,75,76,77].

In this study, we tested for membrane-associated PCNA expression in MM cell lines and in primary BM mononuclear cells from MM patients, employing the 14-25-9 mAb. MM lines and primary cells showed variable levels of cell-membrane PCNA that correlated with the degree of enhancement of NK cell antitumor function following incubation with the 14-25-9 mAb.

## 2. Results

We have shown in several previous studies that the membrane PCNA presents an immune checkpoint, inhibiting NK cells’ functions through the NKp44 splice-variant 1 isoform [31]. In order to interfere with this immune checkpoint and restore NK cell function against PCNA expressing cancer cell lines, we generated a monoclonal antibody that blocks NKp44-PCNA interaction, by binding membrane expressed PCNA. However, the functionality of mAb 14-25-9 in the context of multiple myeloma was not yet tested.

### 2.1. mAb 14-25-9 Detects Membrane PCNA on the Surface of Multiple Myeloma Cell Lines, and on Primary Myeloma Cells

**Figure 1:**
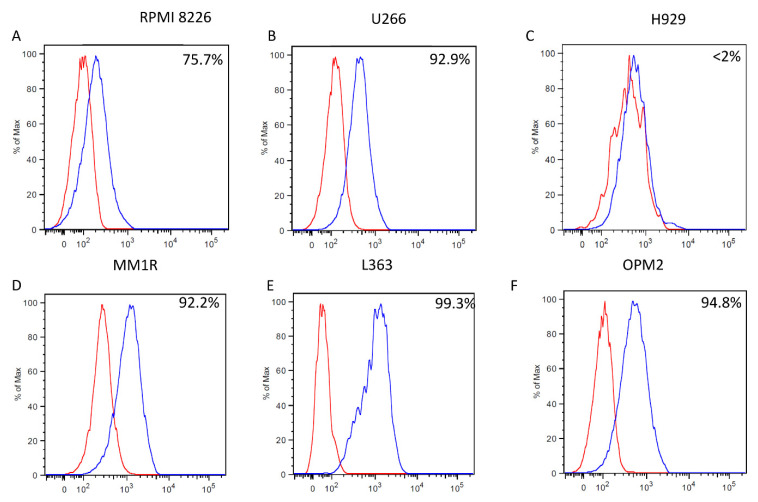
Expression level of cell surface PCNA on different multiple myeloma cell lines. The six different multiple myeloma cell lines indicated (**A**–**F**) were stained with anti-membranal PCNA antibody mAb 14-25-9 (blue line) and mIgG1 control antibody (red line). The cell-bound signal was measured by flow cytometry and analyzed using FlowJo v10. Data are presented from three independent biological replicates and at least two independent experiments. Percentages indicate the fraction of cells positive for membranal PCNA.

**Figure 2:**
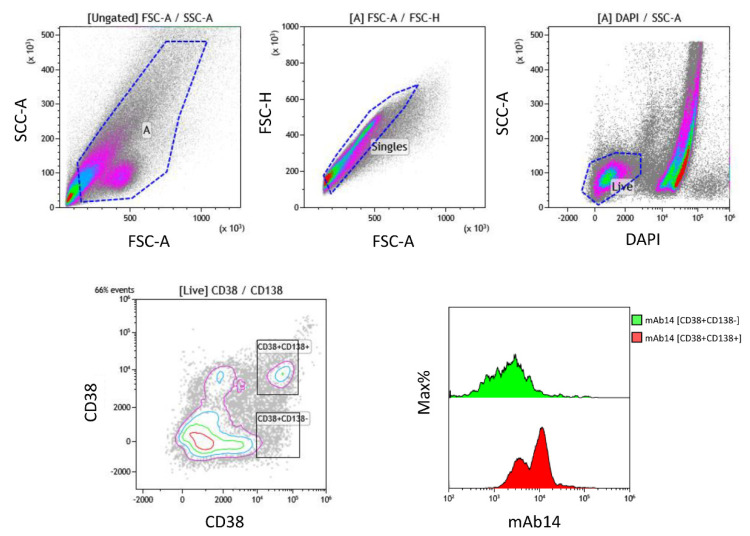
PCNA is expressed on the surface of primary BM mononuclear cells from MM patients. Representative FACS data of the MM-containing cell population (CD38^+^CD138^+^) from the bone marrow of patient #5. As described in the Section 3, patients’ bone marrow aspirates were collected to isolate mononuclear cells. DAPI was used to discriminate between live and dead cells. mAb 14-25-9 employed in this staining was chimeric 14-25-9 in which murine Fc was replaced with human IgG1 Fc to allow multicolor staining with directly labeled murine Abs against CD38 and CD138. Representative data shown is of one out of five patients.

Firstly, we stained six human multiple myeloma cell lines, namely: RPMI8266, U266, L363, OPM2, MM.1R, and H929. We used the mAb 14-25-9 followed by a secondary fluorescent reagent, along with 7AAD to allow the exclusion of dead cells. We observed a significant binding of mAb 14-25-9 to five of the six multiple myeloma cell lines at various levels with the highest staining on the L363 line, followed by U266, OPM2, and MM1R with a medium level of staining, RPMI266, which showed a low level of staining, and H929 that lacked any staining (Figure 1). Mouse IgG1 was used as an isotype control since mAb 14-25-9 was previously determined to be an IgG1 isotype. We then stained primary BM mononuclear cells from multiple myeloma patients to determine their expression of CD138 and CD38 markers along with mAb 14-25-9 staining. We observed that CD38^+^CD138^+^ cell populations were clearly stained with mAb 14-25-9 at various levels. Note that not all CD38^+^CD138^+^ cells stained positively with mAb14-25-9, as compared to CD38^+^CD138^−^, representing non-malignant plasma cells (Figure 2).

### 2.2. PCNA Is Expressed by All Various Plasma Cells from MM Bone Marrow

**Figure 3:**
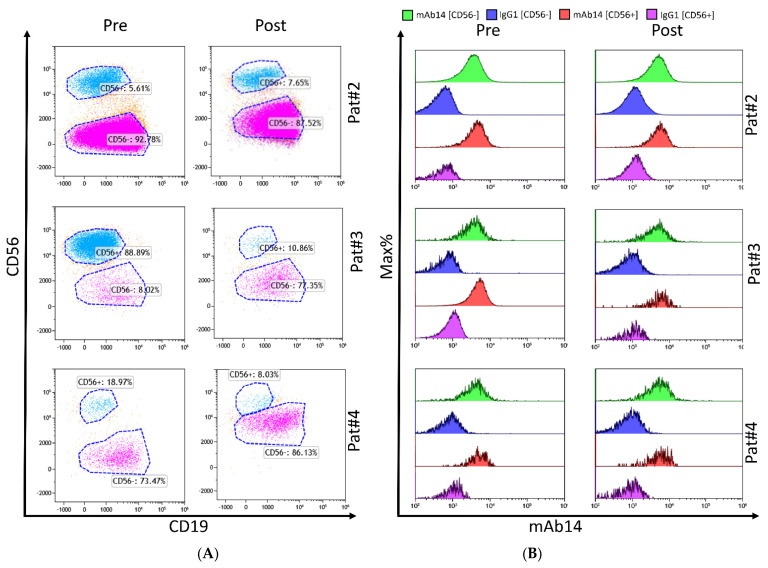
PCNA is expressed on CD38^+^CD138^+^ cells from BM mononuclear cells of MM patients, pre- and post-treatment. For three MM patients (Pat#2, Pat#3, and Pat#4), plasma cells were gated as CD38^+^CD138^+^ and then further dissected by CD56 and CD19 expression. Surface PCNA expression was assessed as a histogram on the CD56^+^CD19^−^ and CD56^−^CD19^+^ subpopulations before (Pre, left) and after (Post, right) treatment. (**A**) Dot plots delineating CD56^+^ (blue) and CD56^−^ (purple) MM cells. (**B**) Overlay histograms of surface PCNA staining (mAb 14-25-9, green and red) compared to the control staining (hIgG1, blue and purple) for both subpopulations (CD56^+/−^). Note that Mab 14-25-9 employed in this staining was chimeric 14-25-9 in which murine Fc was replaced with human IgG1 Fc to allow multicolor staining with directly labeled murine Abs against CD138, CD38, CD56, and CD19. DAPI was used to discriminate against dead cells. Data is shown from three independent biological replicates. Analysis and plots were done using Kaluza software.

With the aim of investigating the nature of PCNA expression on various plasma cells, and MM clones within the bone marrow of individual patients, we stained freshly isolated primary BM mononuclear cell samples pre- and post-treatment from MM patients; cells were stained with markers designed for immunophenotyping neoplastic plasma cell populations (CD56, CD19). We found that while clonal composition may change between patients and pre/post treatment, the staining of PCNA was consistent (Figure 3). Both CD56^−^ and CD56^+^ cells stained positively with mAb 14-25-9 (Figure 3).

### 2.3. mAb14-25-9 Blocking of Membrane PCNA Can Enhance NK Cells Activities against Multiple Myeloma Cells

**Figure 4:**
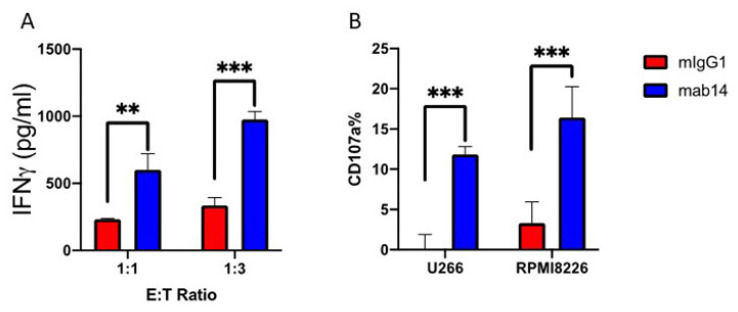
Blocking the membranal-PCNA on MM cell lines enhances IFN-γ secretion and degranulation of NK cells. (**A**) Primary NK cells from a healthy donor were incubated with U266 cell lines and mAb 14-25-9 or mIgG1 for 18 h using different ratios of effector:target (1:1 and 1:3). IFN-γ was measured by ELISA. (**B**) Primary NK cells from a healthy donor were incubated either with RPMI 8226 or U266 cell lines and mAb 14-25-9 or mIgG1 for 5 h. Surface CD107a was measured by flow cytometry, to detect NK cells that had degranulated. Mouse IgG1 acts as a background control (red bars) in comparison to mAb 14-25-9 (blue bars). Dead cells were discriminated against using 7-AAD. Data is shown from three independent replicates and the experiment was repeated three times. Two-way ANOVA test showed *p* ** < 0.01 and *p* *** < 0.001. Statistical analysis and design were performed using Prism GraphPad v8 (California).

Previously we have shown the effect of mAb 14-25-9 as an immune checkpoint inhibitor on various cancerous cells [26,31], to investigate the potency of the mAb 14-25-9 as an immune checkpoint inhibitor in the context of multiple myeloma, we employed several functional assays designed to quantify different indicators of NK cell activation. To this end, we extracted whole blood from a healthy donor and enriched primary NK cells using a commercial negative selection kit. Cells were then cultured for one week in a growth medium supplemented with recombinant human IL2 and used in an experimental model system. Representative multiple myeloma target cell lines were selected (U266 and RPMI8266) and plated on 96 well plates. Target cells were then treated with either mAb 14-25-9 or mouse IgG1 isotype control for one hour on ice, and primary NK cells were then introduced to target cell-bearing wells and incubated for 16 h. Assay media were then collected and secreted IFN-γ was quantified by ELISA. We observed a sharp increase in IFN-γ levels in assay media sampled from wells bearing mAb 14-25-9 treated U266 cells. A dose-dependent increase in total IFN-γ concentration was apparent when the effector:target ratio was tripled in the favor of target cells, which maintained a magnitude of an approx. 3-fold increase in IFN-γ concentration in mAb 14-25-9 treated wells compared to isotype control in both ratios (Figure 4A). The increase in NK activity could not be attributed to the triggering of NK-expressed CD16 since mAb 14-25-9, employed for these functional assays, has murine IgG1 Fc that does not bind to human CD16.

To further validate this finding, we measured primary NK cell degranulation as indicated by the appearance of the CD107a marker on the plasma membrane after exposure to MM cell lines ±mAb 14-25-9. Both U266 and RPMI8266 multiple myeloma cell lines were chosen as target cells. Target cells were plated and treated with either mAb 14-25-9 or mouse IgG1 isotype control similarly to the procedure previously described here. Primary NK cells were then introduced into wells containing the treated cell lines and incubated for 4.5 h. The cells were harvested and stained with anti-human CD107a, CD16, and 7AAD. Dead cells were excluded, and primary NK cells were distinguished from target cells by gating on CD16+ cells. In accordance with trends observed in IFN-γ quantification, we observed an increase in degranulation of the primary NK cells that were incubated with mAb 14-25-9 treated target cells, compared to those incubated with mouse IgG1 isotype control (Figure 4B) in combination with both U266 and RPMI8266 cell lines.

Horton et al. published that PCNA and MHC class I are colocalized to the cell membrane of leukemic cells and that W6/32 fully blocked the binding of NKP44 (that binds membrane-associated PCNA) to the membrane of these cells [78]. The MM cell lines employed in this study are all expressing MHC class I (Supplemental Figure S1A,B, for U266 and RPMI8226 and data not shown). NK92-44-1 cells express high levels of NKp44-isoform-1 that its interaction with membrane-associated PCNA suppresses NK function [31]. Application of either W6/32 or mAb 14-25-9 enhanced the function of NK92-44-1 incubated with U266 or with RPMI8226; application of both antibodies showed an additive effect (Supplemental Figure S1C). As a negative biological target control, we tested the 721.221 cells that lack expression of MHC class I and have no expression of membrane-associated PCNA since they were not stained by mAb 14-25-9. Application of mAb 14-25-9, W6/32, or both antibodies to NK92-44-1 incubated with 721.221 cells did not affect NK function (Figure 1D), showing that the NK activation observed for W6/32 and mAb 14-25-9 was specific to the target expression of cell-surface MHC-I and PCNA, respectively. Thus, mAb 14-25-9 does not exert its enhancement effect through MHC class I. Note that since NK92-44-1 lacks expression of CD16, the effects of the antibodies applied could not be attributed to the triggering of NK-expressed CD16.

To further test the effect of NKp44-1 interaction with membrane-associated PCNA, we tested the membrane levels of NKp44 following co-culture with either membrane PCNA-positive or negative target cells. Four hours incubation of NK92-44-1 cells with membrane PCNA-positive U266 and RPMI8226 resulted in a significant reduction of membrane staining of NKp44. Yet, the same incubation with membrane PCNA-negative target cells did not mediate the reduction of membrane NKp44 on NK92-44-1 cells (Supplemental Figure S2).

### 2.4. mAb 14-25-9 Enhances NK Cells Activities against Primary Multiple Myeloma Cells

**Figure 5:**
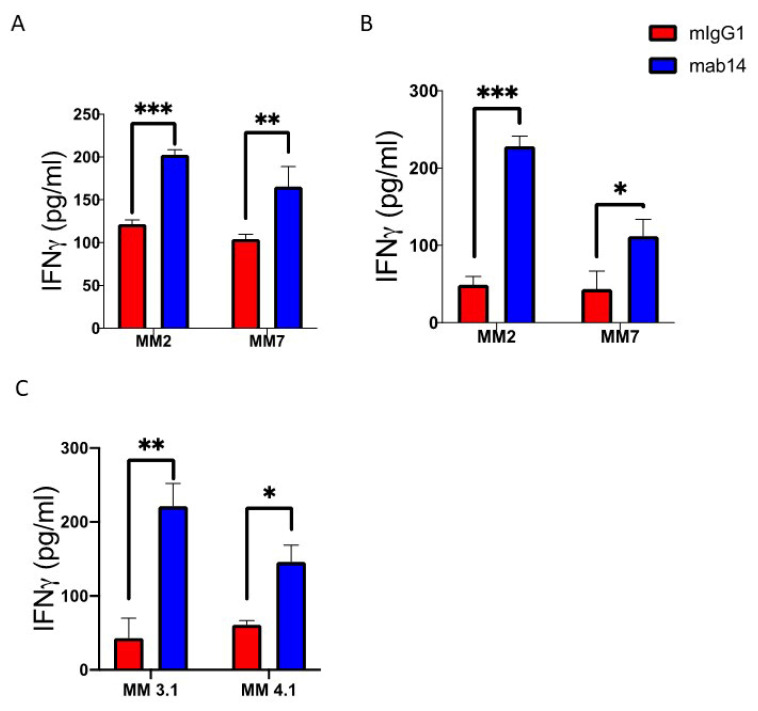
Blocking the membranal-PCNA on primary BM mononuclear cells from MM patients enhanced the activation of NK cells. (**A**) NKp44 isoform 1-transduced NK92 cells (NK92-44-1) were incubated either with Pat#MM2 or Pat#MM7 primary cells for 18 h. (**B**) Primary NK cells from a healthy donor were incubated either with Pat#MM2 or Pat#MM7 primary cells for 18 h. (**C**) Primary NK cells from another unrelated healthy donor were incubated either with Pat#MM3.1 or Pat#MM4.1 primary cells for 18 h. Mouse IgG1 was used as a background control (red bars) for mAb 14-25-9 (blue bars). IFN-γ was measured by ELISA. Data is shown of three technical replicates. Two-way ANOVA test showed *p* * < 0.05, *p* ** < 0.01, and *p* *** < 0.001. Statistical analysis and design were performed using Prism GraphPad v8.

Aiming to establish an ex-vivo model system to assess mAb 14-25-9 efficacy in stimulating NK cell antitumor activity, we have employed MM patient-derived bone marrow samples as the source for target cells in NK cell functional assays. Similar to the experimental design previously described, we plated cells from multiple myeloma patient-derived bone marrow samples on 96 well plates, samples were treated with either mAb 14-25-9 or mouse IgG1 isotype control for one hour on ice, and NK92-44-1 or primary NK cells were then introduced to target cell-bearing wells and incubated for 16 h. IFN-γ was quantified using a standard ELISA assay. When NK92-44-1 was added to primary bone marrow target cells from two different MM patients, the addition of mAb 14-25-9 significantly increased IFN-γ levels in assay media, as compared to the addition of control mouse IgG1 (Figure 5A). Furthermore, the addition of mAb 14-25-9 also increased the secretion of IFN-γ from primary NK cells from two healthy donors when tested with each of these two bone marrow samples from MM patients, as compared to the addition of mouse IgG1 (Figure 5A,B). Moreover, IFN-γ levels positively correlated to mAb 14-25-9 staining levels on MM target cells with a 4.8-fold increase in IFN-γ level in media sampled from pNK cells incubation with Pat#2 target cells (91.7%, gated from CD38^+^CD138^+^ cells, positive for mAb 14-25-9 staining) versus a 2.6-fold increase in media sampled from pNK cells incubation with Pat#7 target cells (30.4%, gated from CD38^+^CD138^+^ cells, positive for mAb 14-25-9 staining) (Figure 5B,C).

## 3. Materials and Methods

### 3.1. Tissue Culture and Cells

The cells we used were RPMI 8266 (ATCC CRM-CCL-155), U266 (ATCC TIB-196), H929 (ATCC CRL-9068), MM.1R (ATCC CRL-2975), OPM-2 (DSMZ ACC50), and L-363 (DSMZ ACC 49). We used RPMI with 10% fetal calf serum (FCS) (Gibco, 12657-029, New York, NY, USA), supplemented with Glutamine, Pen-Strep, non-essential amino acids, 10 mM HEPES, and sodium pyruvate (all from Biological Industries, Israel, Kibbutz Beit-Haemek). NK92 cells, transduced with the canonical NKp44 (splice variant 1, NM_004828.3), and named NK92-44-1 were cultured as previously described [29].

### 3.2. Primary NK Cell Purification

Cells were isolated using a human negative selection-based NK cell isolation kit (EasySep-19615, STEMCELL). Purified NK cells were then cultured in stem cell serum-free growth medium (CellGenix GMP SCGM, 20802-0500) supplemented with 10% heat-inactivated human AB plasma from healthy donors (SIGMA, male AB, H-4522, Israel, Jerusalem), 1% l-glutamine, 1% Pen-Strep, 1% sodium pyruvate, 1% MEM-Eagle, 1% HEPES 1M, and 300 IU/mL recombinant human IL-2 (PeproTech, 200-02-500UG, Cranbury, NJ, USA).

### 3.3. Stimulation Assay and Measure of IFN-γ

To measure NK secreted IFN-γ, the target cells and effector cells were counted; we co-cultured 50,000 cells per well (of microwell 96U-shape plate). Then, 10 µg/mL of mAb 14-25-9, or control mIgG1, was added to individual wells and continued to 18 h culture in a 37 °C, 5% CO_2_ incubator. The secreted IFN-γ were measured using the ELISA MAX Assay (BioLegend, 430116, San Diego, CA, USA), according to manufacturer protocol.

### 3.4. NK Cell Degranulation CD107a Assays

To quantify NK cell degranulation, we first labeled target cells by CFSE (Invitrogen, C34554, Carlsbad, CA, USA).

Both effector and target cells were washed in complete RPMI medium. Then, we recounted each cell type and co-cultured at 50,000 cells per 96U well. We added 10 µg/mL of mAb 14-25-9, or mIgG1, to the experimental wells. To analyze NK activity, we performed a CD107a-degranulation assay as described [79]. Briefly, following incubation of 5 h at 37 °C in a 5% CO_2_ incubator, the percentage of degranulation of effector NK cells was measured by staining for membrane-associated CD107a (SouthernBiotech, H4A3 mAb, Birmingham, AL, USA); the level of CD107a molecule on the cell membrane points to the process of fusion to the cell membrane of NK lytic vesicles.

### 3.5. Flow Cytometry

We washed cells in RPMI medium, then with PBS 1X, and re-counted and plated some 100,000 cells per 96U well. For MM cell lines: mAb 14-25-9, or control mIgG1, were added to a final concentration of 2 mg/mL and incubated for one and half hours on ice. Then we washed cells and stained them with secondary goat anti-mouse conjugated with APC (Jackson ImmunoResearch Laboratories, West Grove, PA 19390, USA). For primary BM mononuclear cells from MM patients: chimeric 14-25-9 (in which murine Fc was replaced by human Fc IgG1) was employed (with isotype control of human IgG1) due to the multi-color staining with directly labeled antibodies to CD138, CD38, CD56, and CD19. Dead cells were labeled by adding 7AAD (BioLegend, 420404) or DAPI to the resuspension after mAb staining.

### 3.6. Primary Myeloma Samples

Bone marrow mononuclear cells from MM patients that contain primary myeloma cells were isolated using a Ficoll-Hypaque density gradient. Heparinized bone marrow aspirates, obtained from patients as part of routine diagnosis, were diluted in a 1:1 (*v*/*v*) ratio with complete RPMI 1640 culture. We avoided possible excess tissues and clumps by filtration through a 70 µm nylon tissue strainer. Single-cell suspensions were isolated over ficoll (Lymphocyte Separation Medium, MPBio). Centrifugation was at 400× *g* for 30 min at 25 °C, *w*/*o* brakes. Bone marrow mononuclear cells were collected and washed twice with culture medium.

### 3.7. Statistical Data Analysis

We used GraphPad Prism software for the statistical analysis. We used either Student’s *t*-test for pair-wise comparisons, or ANOVA for more than 2 groups. *p* < 0.05 is considered statistically significant.

Significance is indicated as * *p* < 0.05; ** *p* <0.01; *** *p* <0.001; **** *p* < 0.0001.

## 4. Discussion

MM is a relatively common hematologic malignancy, that despite many advanced treatments tends to relapse, and in most cases results in the patient’s death [41]. Like many other types of cancer, MM malignant cells can avoid the immune system by exploiting immune checkpoints [80,81]. Blocking of immune checkpoints by mAb gained impressive success in many cancers [82]. Studies have shown the importance of NK cell activity against MM [83], which strongly supports the use of NK cell-targeted therapies in MM. Here we present the activity of a novel immune checkpoint-targeting mAb by our design, in activating NK cells against MM cells.

As explained in the introduction, we have previously reported the interaction between NKp44 and the PCNA [26]. PCNA expression is high in many cancers [27], and its interaction with NKp44 inhibits the activation of NK cells and the lysis of tumor cells by NK cells; we showed that PCNA is expressed on the cell surface of some solid tumors but not on the cell surface of matched healthy tissues [31]. This interaction serves as an immune checkpoint and thus blocking it will achieve anti-tumor activity. In this study, we show that most of the MM cells that were tested express the PCNA marker on their membrane. We found that five out of six popular MM cell lines express membranous PCNA, as well as primary MM cells from a patient’s bone marrow mononuclear cell samples. This expression of PCNA by the malignant cells enables the opportunity of treating MM by targeting cell surface PCNA and blocking its interaction with NKp44. PCNA is not the only “inhibitory ligand” expressed by MM cells. MHC class I expression by target MM cells is suppressing NK function through NK-expressed KIRs and other NK-expressed inhibitory receptors recognizing polymorphic and/or monomorphic MHC class I molecules (reviewed in [84]). We showed that our MM lines are MHC class I positive; Horton et al. published that PCNA and MHC class I are colocalized to the cell membrane of leukemic cells [78]. Yet, we showed that mAb 14-25-9 does not exert its enhancement effect through MHC class I (Supplemental Figure S1). Thus, we believe that NK function enhancement due to the blocking of target-membrane-associated PCNA by mAb 14-25-9 is not involving MHC-I molecules expressed by target cells. At the NK-intracellular signaling level, both KIR- and NKp44-isoform-1-mediated signals are ITIM-dependent; they are probably additive signals as was shown for NK cell education [85].

MM cells also express ligands to NK activating receptors. We previously looked at levels of expression of NCRs, NKG2D, and DNAM-1 in MM patients in different stages of MM trajectory [86]. In some of the patients, we looked at ligands for NKG2D, DNAM-1, and some other receptors on MM cells in the bone marrow and compared these levels to the expression of the corresponding receptor on NK in blood or bone marrow [86]. The only one that showed such a correlation was a negative correlation between NKG2D expression on CD56 bright NK in blood to levels of ULBP1 on MM cells in the bone marrow. Following the results of this study, additional investigation is needed to compare NKp44-1 isoform expression by NK of MM patients to the levels of membrane PCNA expression by their myeloma cells and following myeloma treatment.

Multiple myeloma is known to be a clonally diverse disease with multiple clones present at diagnosis [87]. Clones and sub-clones of MM are changing throughout disease progression. These changes may vary in membrane protein markers [88,89,90], the form of immunoglobulin produced, and may even differ in physical characteristics such as size and internal complexity [91,92,93]. By flow cytometry analysis of primary MM cells derived from patients before and after treatment, we saw that the levels of some MM-related markers (for example CD56) have declined, probably meaning that the cells that express them were eliminated by the treatment. Notably, both the CD56^+^ and the CD56^−^ showed clear staining with mAb 14-25-9 (Figure 3). Whether CD56 expression by MM cells is associated with virulence is not clearly established; yet, our results show a profound expression of the PCNA on MM cells in the bone marrow, regardless of their CD56 expression and clonality before and after treatment (proteasome inhibition, immunomodulatory imide drugs (IMiDs), and dexamethasone). These important results indicate that mAb 14-25-9-mediated blockade of the immune checkpoint (IC) between PCNA and NKp44 still has the potential to be effective, even in combination with conventional therapy, in promoting NK cells to eliminate a wider portion of the malignant cells including various MM clones.

We used our mAb 14-25-9 to block PCNA, and therefore to block the NKp44-PCNA interaction, and evaluated the response of NK cells to MM cells. We measured a much stronger anti-malignant response of NK cells against MM cells that were treated with the mAb 14-25-9. This response was manifested by the release of IFN-γ, a cytokine that is secreted by activated NK cells, and by the expression of CD107a, a marker for NK cell degranulation. This potentiated function against MM cells was seen both with NK cell lines and with primary NK cells and against MM cell lines as well as primary MM cells. Expectedly, activation of NK cells was also seen against MM cells derived from patients who were already treated. This again highlights the immunotherapeutic potential of interfering with this NKp44-PCNA IC, to induce NK cell-mediated elimination of a wide proportion of malignant cells in MM patients. Although further research is needed to examine the consequences of this IC-blocking in vivo, our results have exciting implications for MM therapy. The blocking of the IC can bring stronger NK cells activity against MM cells, which will even strike malignant cells that are otherwise not affected by the conventional treatment.

## 5. Conclusions

In this study of multiple myeloma, we provided pre-clinical evidence that PCNA is expressed on the cell surface of MM cells and that incubation with mAb 14-25-9 enhances NK activity against MM cells. Blocking the NKp44-PCNA IC in combination with other conventional MM treatments should be further explored in clinical trials to study whether this combination could improve therapeutic outcomes for MM patients.

## Data Availability

The data presented in this study are available on request from the corresponding author.

## Supplementary Materials

The following supporting information can be downloaded at: https://www.mdpi.com/article/10.3390/ijms23094717/s1.
