# Supplementary figures and images for: Blocking the PCNA/NKp44 Checkpoint to Stimulate NK Cell Responses to Multiple Myeloma
Source: Int J Mol Sci. 2022 Apr 25;23(9):4717. doi: 10.3390/ijms23094717 (PMC9105815; doi:10.3390/ijms23094717)

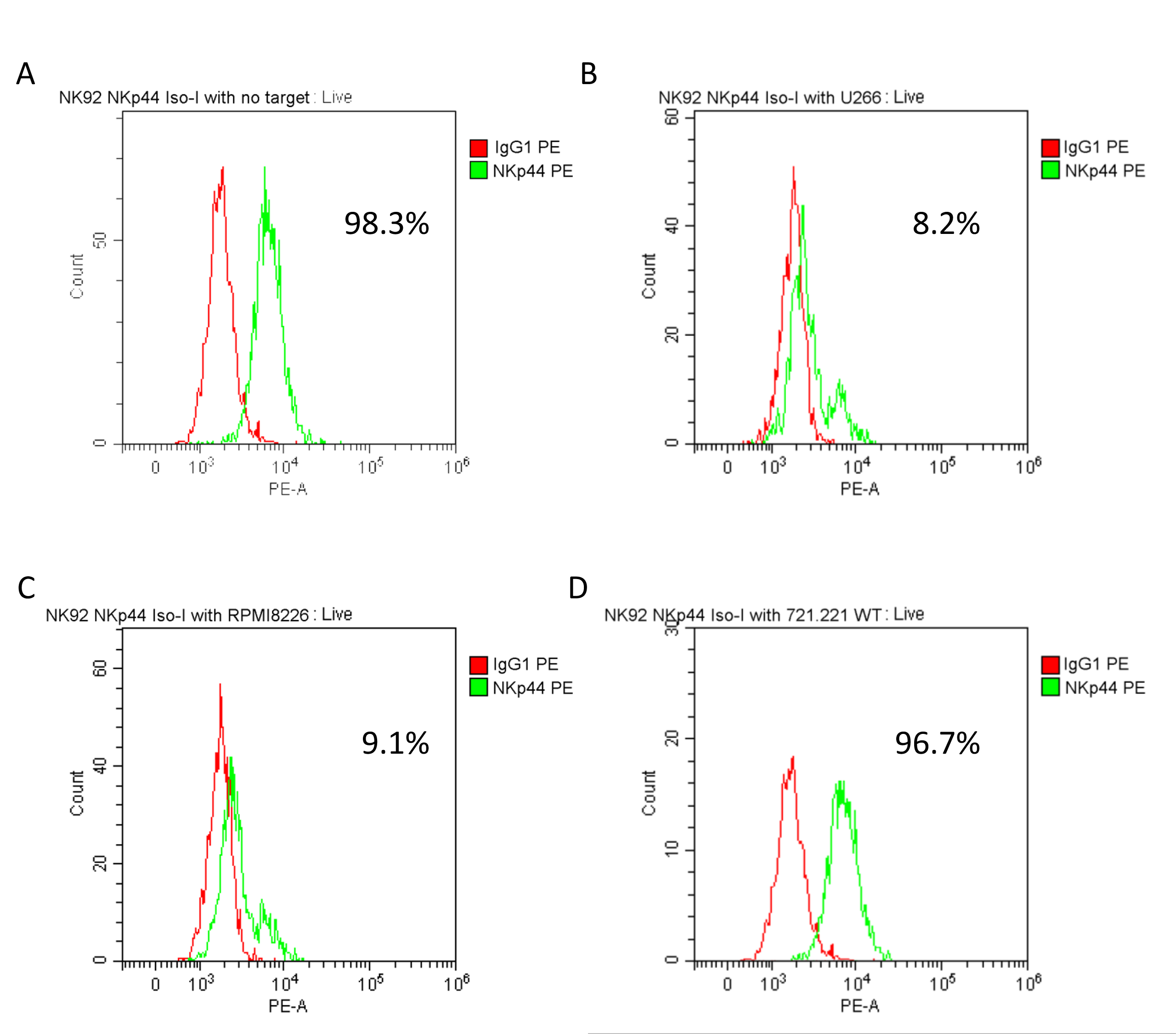

Supplement: Supplementary file 1 [file ijms-23-04717-s001.zip › sup. figure 2.png]

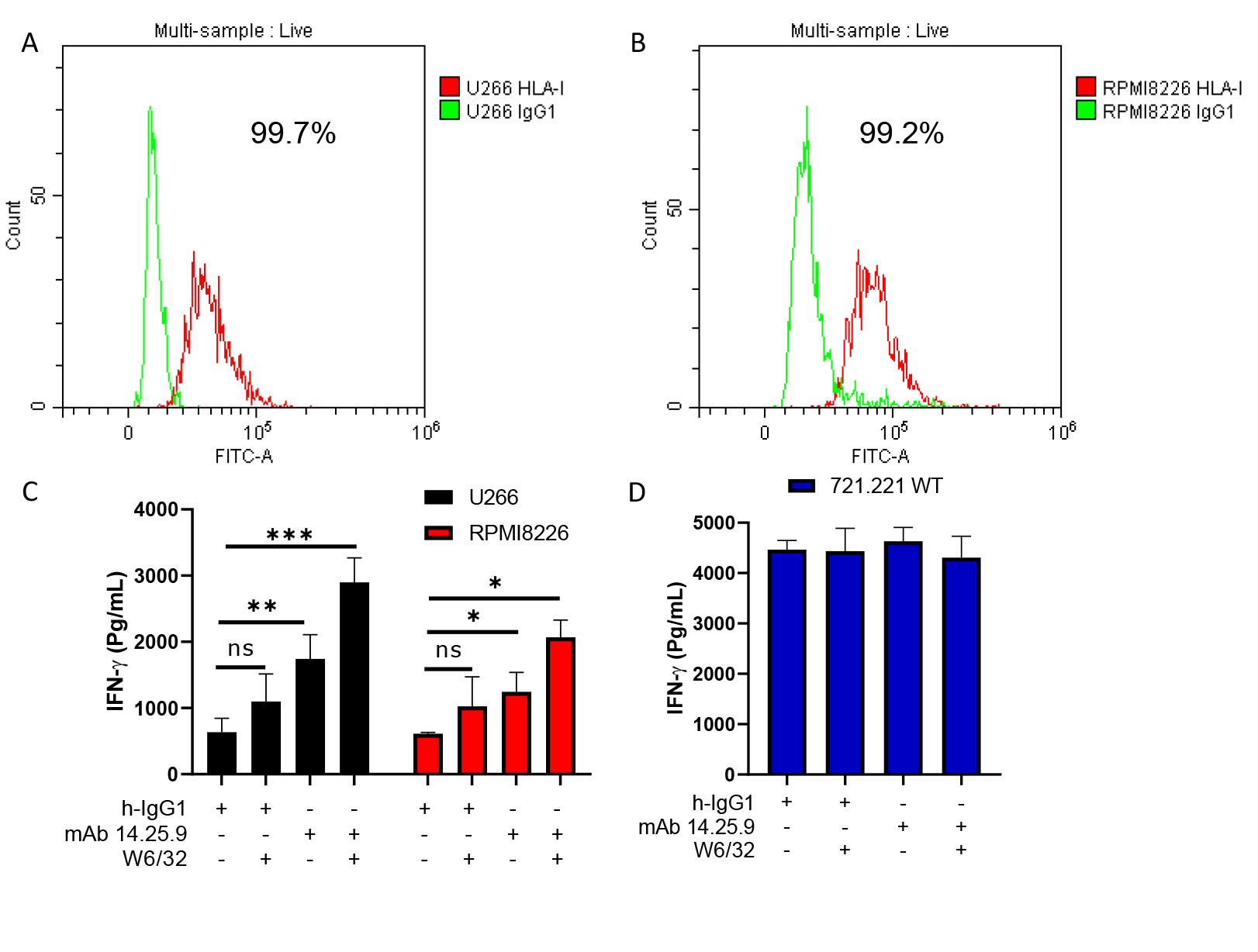

Supplement: Supplementary file 1 [file ijms-23-04717-s001.zip › sup. Figure 1.png]
